# Association of Circulating Plasma Secreted Frizzled-Related Protein 5 (Sfrp5) Levels with Cardiac Function

**DOI:** 10.3390/jcdd10070274

**Published:** 2023-06-28

**Authors:** Conagh J. Kelly, Matthew Chu, Rossana Untaru, Bahador Assadi-Khansari, Dongqing Chen, Amanda J. Croft, John D. Horowitz, Andrew J. Boyle, Aaron L. Sverdlov, Doan T. M. Ngo

**Affiliations:** 1 School of Biomedical Sciences and Pharmacy, University of Newcastle, Callaghan 2308, Australiadongqing.chen@uon.edu.au (D.C.); 2 Hunter Medical Research Institute, New Lambton Heights 2305, Australia; bahador.assadi@gmail.com (B.A.-K.); amanda.croft@newcastle.edu.au (A.J.C.); andrew.boyle@newcastle.edu.au (A.J.B.); 3 School of Medicine, University of Adelaide, Adelaide 5000, Australiajohn.horowitz@adelaide.edu.au (J.D.H.); 4 Hunter New England Local Health District, Newcastle 2305, Australia; 5 School of Medicine and Public Health, University of Newcastle, Callaghan 2308, Australia

**Keywords:** secreted frizzled-related protein 5 (SFRP5), heart failure, cardiovascular function, coronary artery disease, obesity, cardiovascular disease

## Abstract

Secreted frizzled-related protein 5 (SFRP5) is a novel anti-inflammatory adipokine that may play a role in cardiovascular development and disease. However, there is yet to be a comprehensive investigation into whether circulating SFRP5 can be a biomarker for cardiac function. Plasma SFRP5 levels were measured via ELISA in 262 patients admitted to a cardiology unit. Plasma SFRP5 levels were significantly lower in patients with a history of heart failure (HF), coronary artery disease (CAD), and atrial fibrillation (AF; *p* = 0.001). In univariate analyses, SFRP5 levels were also significantly positively correlated with left ventricular ejection fraction (LVEF) (r = 0.52, *p* < 0.001) and negatively correlated with E/E’ (r = −0.30, *p* < 0.001). Patients with HF, CAD, low LVEF, low triglycerides, high CRP, and high eGFR were associated with lower SFRP5 levels independent of age, BMI, or diabetes after multivariate analysis (overall model r = 0.729, SE = 0.638). Our results show that low plasma SFRP5 levels are independently associated with the presence of HF, CAD, and, importantly, impaired LV function. These results suggest a potential role of SFRP5 as a biomarker, as well as a mediator of cardiac dysfunction independent of obesity and metabolic regulation.

## 1. Introduction

Secreted frizzled-related protein 5 (SFRP5) belongs to the secreted frizzled-related protein (SFRP) family that is implicated in multiple cellular processes. These include but are not limited to, acting as extracellular signaling ligands [1], modulating apoptotic events [2], and in early cardiac embryonic development [3]. SFRP5 is primarily secreted by adipocytes, with several studies suggesting it is also secreted by the vascular matrix in adipose tissue [4,5]. It is suggested to be an anti-inflammatory adipokine, binding directly to wingless/integrated (Wnt) family member 5a (Wnt5a), interfering with noncanonical activation of pro-inflammatory pathways in the pathophysiology of obesity-associated metabolic derangements [6]. Specifically, SFRP5 has been shown to be downregulated in the adipose tissue of obese animal models and correlated significantly to impaired glucose and insulin resistance. Conversely, restoration of SFRP5 levels ameliorated obesity-induced glucose intolerance and hepatic steatosis [7].

Recently, data have emerged indicating a role for SFRP5 in regulating cardiac function. For example, there is a downregulation of SFRP5 in cardiac tissue post-myocardial infarction (MI) in mice [8]. Furthermore, SFRP5 knock-out resulted in significantly greater infarct size and infiltration of Wnt5a-positive macrophages into the infarct zone following cardiac ischemia/reperfusion injury (I/R) [9]. Promisingly, restoration of myocardial SFRP5 levels was associated with reduced cardiomyocyte death, minimized myocardial oxidative stress, and a suppressed inflammatory response, which ameliorated the effects of diminished SFRP5 [8].

Currently, the role of circulating SFRP5 levels as a biomarker for cardiovascular diseases (CVD) is not well described, with studies yielding discordant results. The KORA F4 study showed inverse associations between circulating SFRP5 levels and multiple risk factors for type 2 diabetes and CVD, e.g., high BMI, HbA1c, systolic blood pressure, and low HDL cholesterol [10], while others have found that patients with type 2 diabetes had higher SFRP5 levels than patients without diabetes [11].

In this study, we evaluated the relationship between circulating SFRP5 levels and left ventricular systolic and diastolic function in patients with CVD. We also examined whether the relationship between SFRP5 and these parameters of cardiac function is independent of obesity and diabetes.

## 2. Materials and Methods

### 2.1. Study Population

The study population consisted of 262 patients with established cardiovascular disease who were admitted to the cardiology units at the John Hunter Hospital, New South Wales (NSW) or The Queen Elizabeth Hospital, South Australia (SA) in Australia. The study was approved by the Human Research Ethics Committee of Hunter New England Local Health District (reference number: 2018/ETH00125), and all patients enrolled in the study provided written informed consent. The investigation conforms with the principles outlined in the Declaration of Helsinki [12].

Inclusion criteria: Patients admitted as an inpatient under cardiology care at either hospital; age ≥ 18 years; echocardiogram during or within 3 months of admission; able to provide written informed consent. Exclusion criteria: Patients who did not have echocardiography performed (or image quality was too poor for assessment of diastolic function); inability to provide written informed consent; age < 18 years.

Patient demographics and comorbidities were collected from patient interviews, electronic or written medical records, and medication chart reviews. Pharmacological treatments were also recorded at the time of admission. Hospitalizations and hospital discharge diagnoses were collected from electronic or written medical records. Comorbidities and admission diagnoses were verified from the ICD-10 (International Statistical Classification of the Diseases and Related Health Problems) codes recorded during the hospital admission when the patients were recruited to the study.

Patients underwent routine biochemical analyses of blood as well as measurements of anthropometric parameters.

### 2.2. Echocardiography

Echocardiographic measurements were performed in accordance with the American Society of Echocardiography and European Association of Cardiovascular Imaging guidelines [13]. M-mode echocardiographic analysis was used to assess left ventricular (LV) interventricular septal dimensions (IVSd) and LV posterior wall thickness. Left ventricular ejection fraction (LVEF) was assessed via the modified Simpson’s method in a biplane using 2D images. The mitral flow velocity pattern was utilized to determine early diastolic filling (E) and peak filling velocity at atrial contraction (A). Tissue Doppler was used to assess peak mitral annular velocity during early (E’) and late (a’) filling. All measurements were averaged over 3 consecutive cardiac cycles.

### 2.3. Blood SFRP5 Measurements

All blood samples were collected via venipuncture, centrifuged to separate plasma from whole blood, and kept at −80 °C until SFRP5 analysis. The plasma concentrations of SFRP5 were analyzed using a commercially available enzyme-linked immunosorbent assay (ELISA) kit (Cloud Clone Corp., Houston, TX, USA). The ELISA was performed as per the manufacturer’s instructions.

### 2.4. Statistical Analysis

Normally distributed continuous variables are shown as mean ± standard deviations (SD), whereas non-normally distributed continuous variables are presented as a median (min, max). Categorical variables are presented as numbers (percentage (%)) unless otherwise stated. Comparisons between groups were performed using the unpaired *t*-test or the Mann–Whitney U-test, as appropriate. Data that were not normally distributed were logarithmically transformed before linear regression analysis. Relationships between SFRP5 levels and patient characteristics (age, gender, BMI, and diabetes) and parameters significant on univariate analysis (CAD, HF, AF, dyslipidaemia, statin use, E/E’, high sensitivity c-reactive protein (CRP), LVEF, triglycerides, and eGFR) were assessed via multivariable analyses using backward linear regression analysis. All statistical analyses were performed using SPSS 27 (IBM Corp., New York, NY, USA), and *p* < 0.05 was considered statistically significant.

## 3. Results

### 3.1. Characteristics of Study Cohort

Baseline demographic and clinical characteristics are summarized in Table 1. One hundred fourteen patients (43.5%) were female. The mean age of the participants was 68 ± 11 years, with a median BMI of 28.4 kg/m^2^. A total of 80 patients (30.5%) had heart failure, 133 patients (50.8%) had coronary artery disease, and 48 patients (18.3%) had atrial fibrillation. Hypertension was the most common comorbidity (*n* = 148; 56.5%).

### 3.2. Univariate Analyses

**Figure 1:**
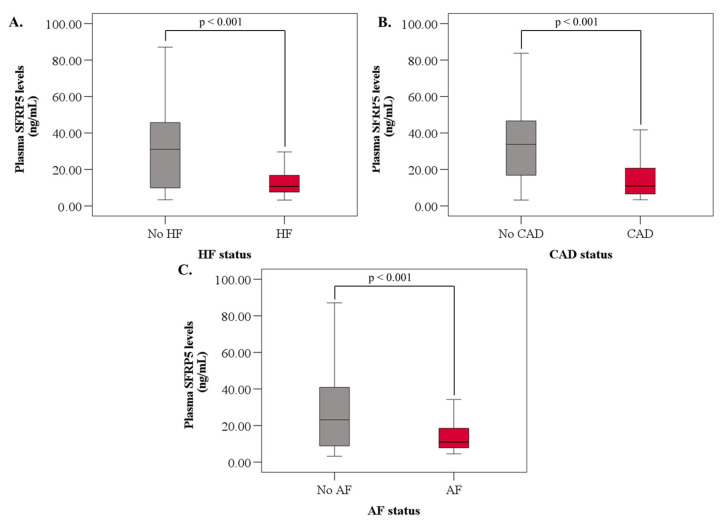
Relationship between SRFP5 levels and CVD states. SFRP5 levels are lower in patients with (**A**) HF, (**B**) CAD, and (**C**) AF. The horizontal lines within the boxes represent the median values, the upper and lower limits of the box represent the 75th and 25th percentiles, respectively, and the upper and lower whiskers represent the minimum and maximum after removing outliers. *p* < 0.05 is considered significant. HF: heart failure, CAD: coronary artery disease, and AF: atrial fibrillation.

SFRP5 levels were significantly lower in patients with HF (10.7 [3.2, 35.7] vs. 31.0 [3.4, 87.1], *p* < 0.001), CAD (11.0 [3.4, 87.1] vs. 33.8 [3.2, 83.8], *p* < 0.001), and AF (11.2 [4.6, 82.0] vs. 23.2 [3.2, 87.1], *p* = 0.001) (Table 2; Figure 1A–C). There was no difference in SFRP5 levels between males and females. Additionally, patients with diabetes or hypertension did not have significantly different SFRP5 levels vs. those who did not have diabetes or hypertension, respectively. Patients with dyslipidemia had significantly higher (28.5 [3.2, 87.1] vs. 11.1 [3.4, 83.8], *p* < 0.001) SFRP5 levels, whilst statin use was associated with lower SFPR5 levels (14.5 [3.2, 87.1] vs. 27.7 [3.4, 83.8], *p* < 0.001). The use of angiotensin-converting enzyme inhibitors/angiotensin receptor blockers (ACEI/ARBs) was associated with a non-significant trend towards lower plasma SFRP5 levels (16.6 [3.9, 83.8] vs. 22.2 [3.2, 87.1], *p* = 0.08) (Table 2).

**Figure 2:**
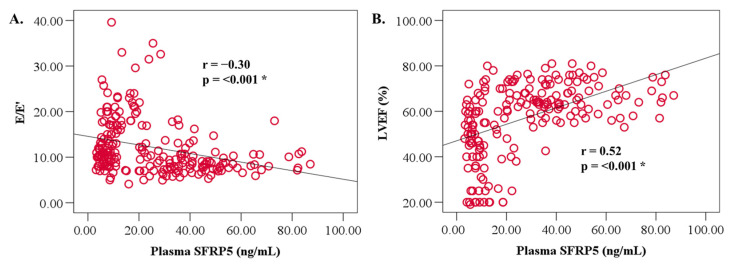
Relationships between SFRP5 and parameters of cardiac (**A**) diastolic [E/E’] and (**B**) systolic [LVEF] function. The dots represent individual patient parameters, and the solid line is a line of best fit. *p* < 0.05 is considered significant (*). LVEF; left ventricular ejection fraction.

SFRP5 levels correlated significantly and directly with EF (r = 0.52, *p* < 0.001) (Figure 2A) and inversely with E/E’ (r = −0.30, *p* < 0.001) (Figure 2B). No relationships were observed between SFRP5 levels and age, BMI, and gender (Table 3). Furthermore, total cholesterol and triglyceride levels correlated directly with SFRP5 levels (r = 0.29, *p* < 0.001; r = 0.17, *p* < 0.01, respectively), while CRP levels were correlated inversely with SFPR5 levels (r = −0.29, *p* < 0.001) (Table 3). Moreover, eGFR was significantly directly correlated with plasma SFRP5 levels (r = 0.16, *p* = 0.02) upon univariate analysis (Table 3).

### 3.3. Multivariable Analyses

Upon multivariable analyses (adjusted for age, gender, BMI, diabetes, CAD, HF, AF, dyslipidaemia, statins, E/E’, LVEF, triglycerides, eGFR, and CRP), the significant predictors of lower plasma SFRP5 levels were CAD (β = −0.36, *p* = 0.019), HF (β = −0.64, *p* = <0.001), statin use (β = −0.24, *p* = 0.050), CRP (β = −0.12, *p* = 0.010), and eGFR (β = −0.01, *p* = 0.014) (Table 4), while LVEF (β = 0.12, *p* = 0.016) and triglyceride levels (β = 0.24, *p* = 0.042) were associated with higher SFRP5 levels, as shown in Table 4.

## 4. Discussion

Our study demonstrates the robust downregulation of circulating SFRP5 levels in both heart failure and CAD, independent of obesity and diabetes. We also show that SFRP5 levels are associated with both systolic and diastolic cardiac dysfunction: low SFRP5 is associated with (1) low LVEF and (2) elevated E/E’. These results highlight the potential role of SFRP5 as a cardiovascular biomarker, as well as its likely role in regulating cardiac function.

The role of SFRP5 levels as a biomarker for metabolic perturbations has been investigated in several population-based observational studies. The largest of these studies demonstrated that serum SFRP5 levels are inversely associated with multiple cardiometabolic risk factors, including BMI and type 2 diabetes [10], thereby confirming the relationship between low SFPR5 levels and cardiometabolic risk factors. On the other hand, Du et al. found that while serum SFRP5 levels were negatively associated with BMI and type 2 diabetes in the control population of their study, these relationships were absent in patients presenting with acute myocardial infarction [14]. Consistently, in the current study, we observed no relationships between SFRP5 levels and BMI or diabetes status in patients with established CVD. Our data instead suggest that HF and CAD are strong determinants of SFRP5 levels, potentially outweighing the effects of individual metabolic perturbations, which in themselves may play a role in the development of CVD, resulting in CVD being a more “overarching” predictor of SFRP5 levels.

Numerous studies have demonstrated that SFRP5 is an anti-inflammatory adipokine with direct effects in regulating systemic metabolic homeostasis [7,15]. The effects of SFRP5 in regulating cardiac function are less known, with only several studies examining the role of SFRP5. Circulating SFRP5 levels were significantly lower in patients with CAD and concomitant HF vs. those with CAD alone, while low SFRP5 levels also correlated with low LVEF in these patients, suggesting a dynamic interdependent relationship [16]. Wu et al. found that higher SFRP5 levels were associated with a better HF prognosis in a cohort of HF patients [16]. In the present study, we observed that SFRP5 levels are downregulated in patients with diagnosed HF compared to those without. The additional novel finding in the current study is that SFRP5 levels were significantly associated with both HF as a diagnosis, as well as EF per se, independent of potentially confounding variables, including the presence of CAD and major CV risk factors. Thus, SFRP5 levels could be used to separate HF from non-HF patients in a heterogeneous cohort of CVD patients confirming that it may be suitable for diagnostic as well as risk stratification purposes in HF patients [16].

One of the most salient findings of the current study is the observed relationship between SFRP5 and diastolic cardiac function: this is the first study to demonstrate this. E/E’ is elevated in patients with diastolic dysfunction and is one of the main echocardiographic parameters used for the diagnosis/evaluation of heart failure with preserved ejection fraction (HFpEF) [17]. HFpEF accounts for up to 50% of HF cases and is associated with high morbidity and mortality. Conventional HF therapies are ineffective in HFpEF, with the exception of SGLT2 inhibitors, which interestingly are metabolic regulators developed and used for the treatment of diabetes [18]. Furthermore, obesity and metabolic dysfunction, including diabetes, are the main comorbidities that are associated with HFpEF [19], whereas SFRP5 has been previously shown to be influenced by obesity and metabolic dysfunction [6,10,20]. Thus, our observation that downregulated SFRP5 levels were associated with elevated E/E’ may suggest a new molecular pathway underlying the nexus between obesity/metabolic syndrome and diastolic dysfunction/HFpEF. However, more in-depth population and mechanistic studies are required to confirm this relationship.

Our data show that patients with CAD have lower SFRP5 levels is consistent with Tong et al., who also observed reduced circulating SFRP5 levels in CAD patients, which correlated with reduced EF [21]. Our observation that low SFRP5 levels were correlated with elevated hs-CRP is in line with the described anti-inflammatory effects of SFRP5. Mechanistic studies have demonstrated that SFRP5 can inhibit myocardial inflammation and injury following a mouse model of ischemia/reperfusion injury [9], possibly exerting anti-inflammatory action via inhibition of Wnt5a. SFRP5 has also been shown to exhibit vascular protection, restoring Wnt5a-induced endothelial dysfunction and vascular relaxation in human vascular endothelial cells [22].

The limitations of our study include its cross-sectional design, and therefore we are unable to establish a causal link between SFRP5 and the parameters described. In addition, many patients were on various cardiovascular medications, and while we attempted to control for these in the multivariate model, we cannot reject the possible influence of the medical therapies on the observed SFRP5 relationships. Furthermore, this study only included patients with documented CVD; therefore, these data may not apply to the general population. Importantly, we did not have NT-proBNP or troponin levels for all the patients, as the measurements of NT-proBNP or troponin were not clinically indicated for all patients; furthermore, in Australia, inpatient measurements of BNP/NT-proBNP are not reimbursed, and thus, they are seldom performed. Finally, we did not measure Wnt5a, SFRP5’s main molecular target, and therefore we cannot comment on the mechanistic downstream pathways involved in observed relationships.

To conclude, we demonstrated that circulating SFRP5 levels were significantly lower in patients with HF and CAD. The results in HF patients are further reinforced by the relationship between SFRP5 and LVEF as well as E/E’, one of the main echocardiographic parameters used to assess diastolic dysfunction and HFpEF, potentially highlighting a pathway in which metabolic and/or adipose tissue dysfunction affects the heart. Taken together, our study provides first insights into the possible role of SFRP5 as a biomarker, as well as a potential mediator of LV dysfunction; the latter would need to be dissected further in mechanistic studies.

## Figures and Tables

**Table 1:** Baseline patient characteristics.

Grouped Characteristics	*n* = 262
Age (years)	68 ± 11
Males, n (%)	148 (56.5)
BMI (kg/m^2^); median [min, max]	28.4 [16.0, 64.7]
Heart failure, n (%)	80 (30.5)
Diabetes, n (%)	100 (38.2)
Hypertension, n (%)	148 (56.5)
Coronary artery disease, n (%)	133 (50.8)
Dyslipidaemia, n (%)	143 (54.6)
Atrial fibrillation, n (%)	48 (18.3)
Left ventricular ejection fraction (%)	57.2 ± 15.8
E/E’; median [min, max]	10.1 [4.1, 39.6]
Statins, n (%)	142 (54.6)
ACEI/ARB, n (%)	141 (53.8)
SFRP5 (ng/mL); median [min, max]	20.1 [3.2, 87.1]
eGFR (mL/min/1.73 m^2^)	76.6 ± 24.4
CRP (mg/L); median [min, max]	3.7 [0.2, 318.0]
Triglycerides (mmol/L); median [min, max]	1.23 [0.3, 12.3]
Cholesterol (mmol/L)	4.5 ± 1.2

Data are shown as mean ± SD or n (%) unless otherwise stated. BMI: body mass index; ACEI: angiotensin-converting enzyme inhibitor; ARB: angiotensin receptor blocker; CRP: high sensitivity C-reactive protein; SFRP5: secreted frizzled-related protein 5; eGRF: estimated glomerular filtration rate.

**Table 2:** Relationships between SFRP5 and categorical patient variables.

Variable	SFRP5 Level (Median [Min, Max])	*p*
Gender		
Male	18.2 [3.4, 82.0]	0.26
Female	21.2 [3.2, 87.1]	
Hypertension		
Yes	22.5 [3.9, 83.8]	0.11
No	15.2 [3.2, 87.1]	
Diabetes		
Yes	20.59 [4.6, 79.8]	0.59
No	18.49 [3.2, 87.1]	
Dyslipidemia		
Yes	28.5 [3.2, 87.1]	<0.001 *
No	11.1 [3.4, 83.8]	
Statins		
Yes	14.5 [3.2, 87.1]	<0.001 *
No	27.7 [3.4, 83.8]	
ACEI/ARB		
Yes	16.6 [3.9, 83.8]	0.08
No	22.2 [3.2, 87.1]	
HF		
Yes	10.7 [3.2, 35.7]	<0.001 *
No	31.0 [3.4, 87.1]	
CAD		
Yes	11.0 [3.4, 87.1]	<0.001 *
No	33.8 [3.2, 83.8]	
AF		
Yes	11.2 [4.6, 82.0]	<0.001 *
No	23.2 [3.2, 87.1]	

ACEI: angiotensin-converting enzyme inhibitor; ARB: angiotensin receptor blocker; AF: atrial fibrillation; CAD: coronary artery disease; HF: heart failure. *p* < 0.05 is considered significant (*). Independent samples Mann–Whitney U test.

**Table 3:** Univariate relationships between SFRP5 and patient variables.

Variable SFRP5	r	*p*
Age	0.03	0.59
BMI	−0.01	0.88
eGFR	0.16	0.02 *
C-Reactive Protein	−0.29	<0.001 *
Triglycerides	0.17	0.01 *
Cholesterol	0.29	<0.001 *

BMI: body mass index; LVEF: left ventricular ejection fraction; eGFR: estimated glomerular filtration rate. *p* < 0.05 is considered significant (*). Non-normally distributed data were transformed before analysis.

**Table 4:** Relationships between SFRP5 and patient demographics upon multivariate analysis.

Variable	β	*p*
HF	−0.64	<0.001 *
CAD	−0.36	0.019 *
Statin use	−0.24	0.050
LVEF	0.12	0.016 *
Triglycerides	0.24	0.042 *
CRP	−0.12	0.010 *
eGFR	−0.01	0.014 *

The model was able to explain a large proportion of the variance in SFRP5 levels (R = 0.729). Adjusted for age, gender, BMI, diabetes, and variables significant on univariate analysis: (CAD, HF, AF, dyslipidaemia, statins, E/E’, LVEF, triglycerides, eGFR, and CRP). *p* < 0.05 is considered significant (*).

## Data Availability

All data were collected via Hunter New England Health systems. All collected data are available upon request.
